# Integration of Digital Smile Design Into Aesthetic and Functional Crown Lengthening: A Clinical Case Report

**DOI:** 10.7759/cureus.108197

**Published:** 2026-05-03

**Authors:** Divena S Choudhary, Sujeet V Khiste

**Affiliations:** 1 Periodontology, Mahatma Gandhi Mission (MGM) Dental College and Hospital, Navi Mumbai, IND

**Keywords:** aesthetic dentistry, altered passive eruption, computer-aided design/computer-aided manufacturing (cad/cam), crown lengthening, digital dentistry, smile designing

## Abstract

Altered passive eruption is characterized by the position of gingival margins coronal to the cementoenamel junction, resulting in short clinical crowns and excessive gingival display, which is confirmed by clinical and radiographic examination. The present case report describes a 42-year-old systemically healthy female patient who reported with a dislodged prosthesis in the maxillary anterior region and dissatisfaction with her smile aesthetics. A periapical lesion on tooth 13 and altered passive eruption were confirmed by clinical and radiographic examination. Treatment was planned according to the etiology, and a digital mock-up was prepared using computer-aided design/computer-aided manufacturing (CAD/CAM) software, followed by surgical crown lengthening with a 3D-printed guided stent. For a precise initial gingival incision, electrosurgery was used under controlled settings, and a full-thickness flap was elevated for osseous recontouring. Temporary prostheses were delivered immediately postoperatively, and later lithium disilicate restorations were placed on teeth 13-23. Patient satisfaction was achieved, crown height-to-width proportions of the restorations were improved, and the gingival display was successfully reduced by 2 mm while maintaining probing depth within 2 mm. This case demonstrates how collaborative care and digital planning can help patients with altered passive eruption achieve precise aesthetic results.

## Introduction

In the modern world, a smile is an important feature for communication and self-presentation. Excessive gingival display, commonly referred to as a “gummy smile,” can make individuals self-conscious about their appearance. It is an aesthetic disharmony characterized by the display of more than 3 mm of gingiva during a full smile, also known as a high smile line [1]. The American Academy of Periodontology classifies excessive gingival display as a mucogingival deformity [2]. Various treatment modalities are available to improve aesthetic outcomes, depending on the underlying etiology, such as short lip length, hyperactive lip movement, short clinical crowns, dentoalveolar extrusion, altered passive eruption, impaired active eruption, vertical maxillary excess, and gingival overgrowth. To formulate an appropriate treatment plan, the etiology is diagnosed based on intraoral and extraoral clinical and radiographic assessments [3]. These conditions often prompt patients to seek treatment for both aesthetic and functional concerns. Among these, altered passive eruption is characterized by gingival margins positioned coronal to the cemento-enamel junction, resulting in short clinical crowns and excessive gingival display [4]. Cohen classified altered passive eruption and proposed corresponding treatment approaches [5].

Aesthetic dentistry involves the integration of multidisciplinary treatment protocols to achieve predictable and patient-centered outcomes. Based on the classification of type (I/II) and subgroup (A/B) of altered passive eruption, crown lengthening is commonly indicated, as it increases clinical crown height to allow for optimal restorative margins while preserving the surrounding supracrestal tissue for a healthy periodontium. Preoperative assessment is critical, as it guides surgical planning regarding alveolar bone levels and the final gingival margin position after healing [6]. To maintain periodontal health around restored teeth, a minimum keratinized tissue width of 2-3 mm is recommended, as it is associated with improved plaque control. Ingber et al. emphasized that the optimal dimension of the supracrestal tissue attachment should be approximately 3 mm, which is the distance between the alveolar crest and the restorative margin. Although this may vary among patients and their phenotypes, it is necessary for proper healing and re-establishment of the dentogingival complex [7].

To enhance efficiency and precision, digital technologies have been increasingly incorporated into treatment planning. Digital Smile Design (Madrid, Spain) is a valuable tool that enables precise visualization of the anticipated aesthetic outcome by evaluating tooth dimensions and morphology in relation to the surrounding gingival architecture, thereby improving patient communication and satisfaction. Additionally, lithium disilicate restorations demonstrate favorable mechanical properties, including high tensile strength and fatigue resistance [8].

This case report describes a multidisciplinary approach involving digitally guided crown lengthening and prosthetic rehabilitation to enhance smile aesthetics in a patient presenting with altered passive eruption and compromised anterior restorations.

## Case presentation

**Figure 1:**
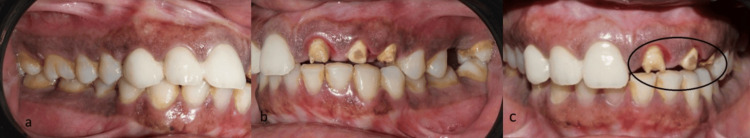
Preoperative images: (a) right lateral view, (b) left lateral view, and (c) frontal view demonstrating the dislodged prosthesis (as marked)

A 42-year-old systemically healthy female patient presented to the Department of Prosthodontics with the chief complaint of recurrent dislodgement of a prosthesis in the maxillary anterior region. Two weeks prior, she experienced dislodgement of the prosthesis involving teeth 21 to 23 during mastication, while the prosthesis from 13 to 11 remained intact. Her dental history revealed multiple root canal treatments followed by repeated prosthetic rehabilitations in the same region over the past eight years. On clinical examination, insufficient clinical crown height of 4-5 mm and excessive gingival display of approximately 2 mm at the central incisors and 3 mm at the lateral incisor and canine regions, along with anterior spacing, were observed (Figure 1). The width of the attached gingiva ranged between 3 and 4 mm, and periodontal probing depths were ≤3 mm, indicating a periodontally healthy status. Altered passive eruption type I-B (Coslet and colleagues’ classification, 1977) was diagnosed based on excessive gingival display and transgingival probing under local anesthesia to determine the bone crest and CEJ levels, supported by radiographic findings. Radiographic evaluation demonstrated a periapical lesion associated with tooth 13, suggestive of reinfection. Based on these findings, endodontic retreatment (re-RCT) was advised.

**Figure 2:**
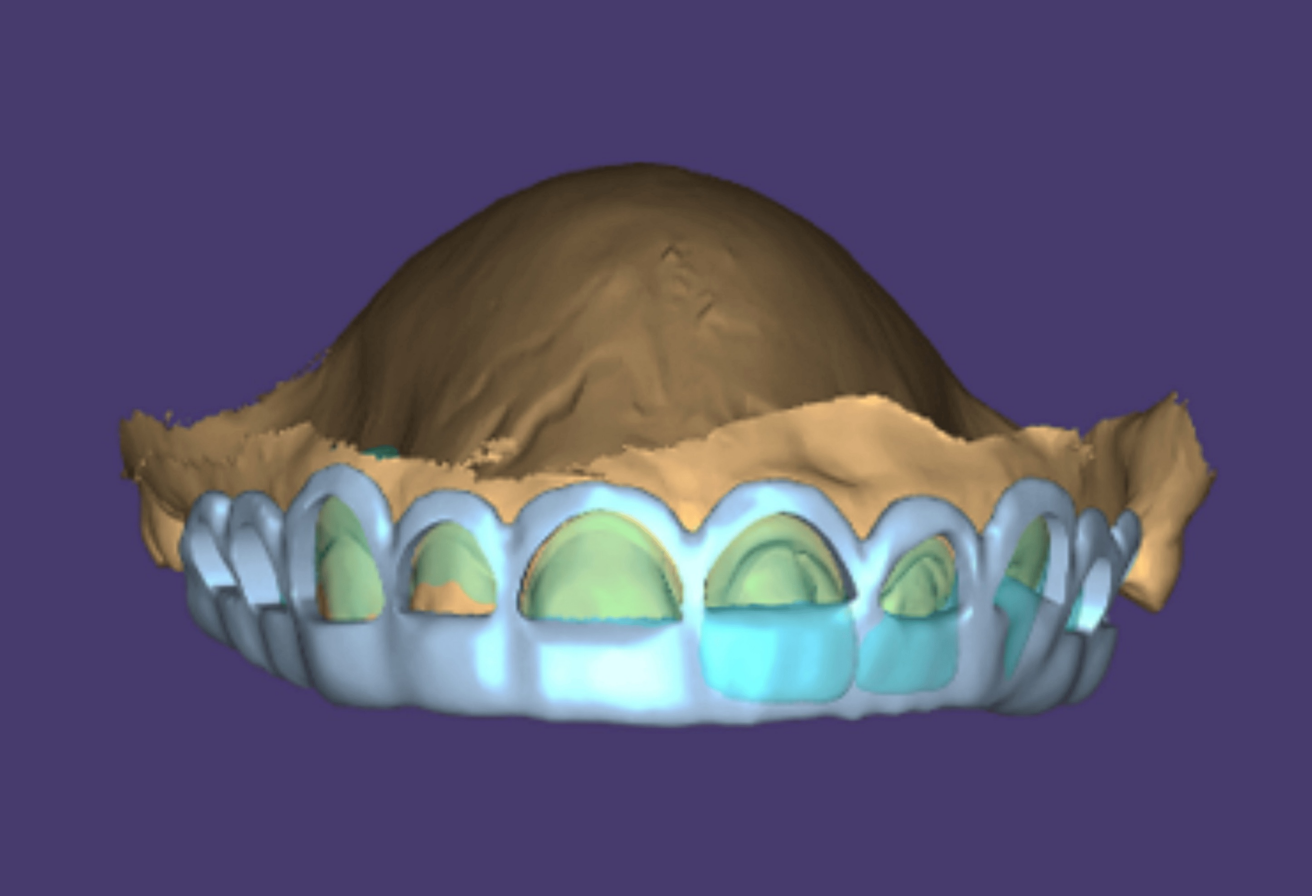
Digital mock-up design finalized after analysis of the patient’s facial features and smile Image Credit: Authors using Digital Smile Design (Madrid, Spain)

The patient expressed significant concern regarding her facial aesthetics, particularly her smile, and reported dissatisfaction due to anterior diastemas and excessive gingival display. Based on the clinical and radiographic findings, a comprehensive treatment plan was formulated, involving surgical crown lengthening in the region of teeth 13-23, followed by definitive full-coverage prosthetic rehabilitation. Prior to the crown lengthening procedure, re-RCT of tooth 13 was performed. At the three-month follow-up, the periapical lesion had resolved. A digital workflow was adopted for treatment planning, using an intraoral scan for soft-tissue guidance and CBCT for evaluation of the bone crest. In conjunction with these scans and clinical assessment of the interpupillary and midfacial lines, a diagnostic mock-up was generated using Digital Smile Design. This was digitally superimposed, and a 3D-printed temporary prosthesis was tried on the patient to facilitate aesthetic evaluation and obtain patient approval (Figure 2). Based on the approved design, a tooth-supported, double-margin, 3D-printed resin surgical stent was fabricated to guide the clinical procedure and accurately determine the positions of the future gingival margins.

**Figure 3:**
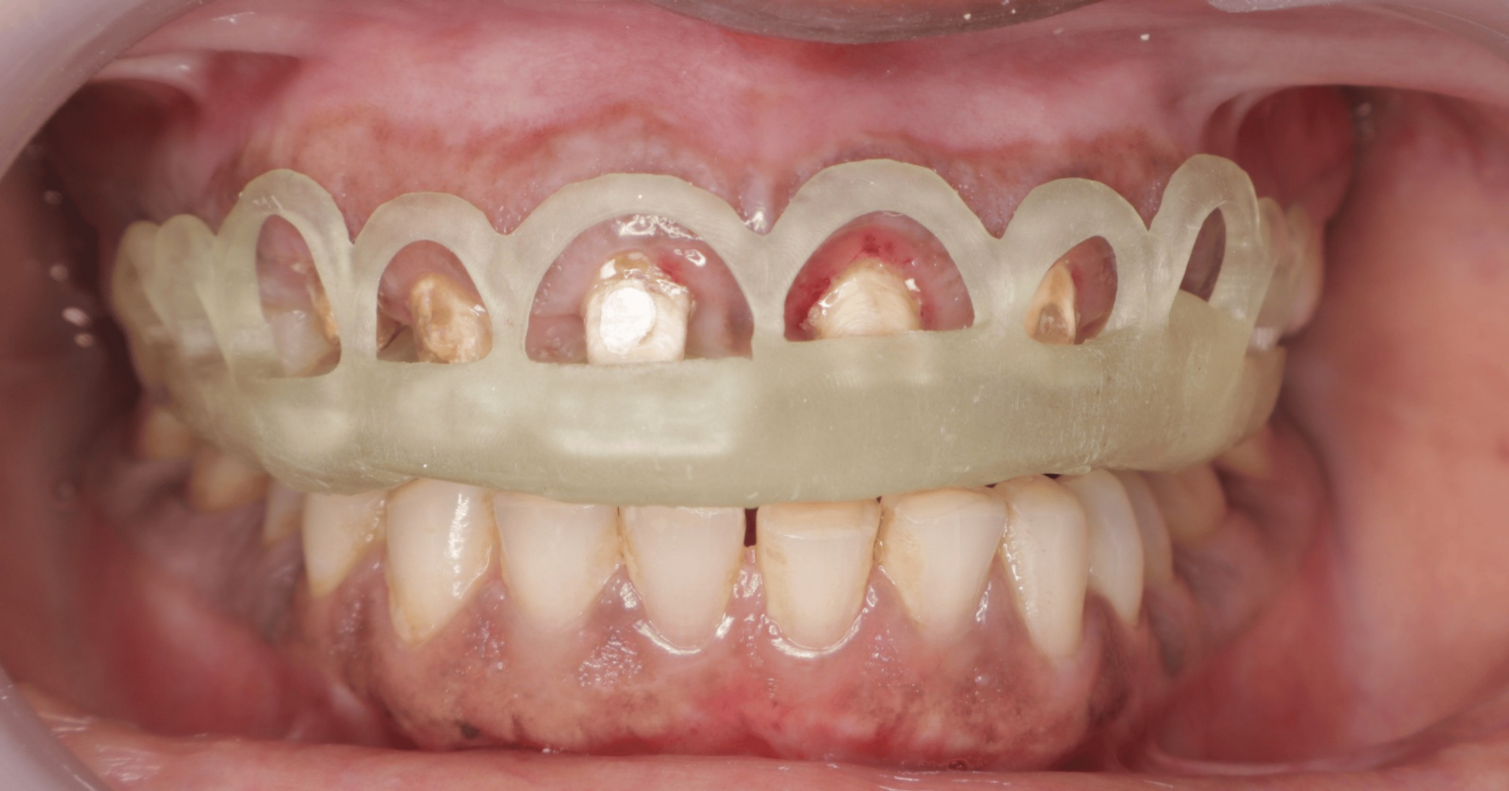
Surgical stent was prepared following approval of the mock-up design

**Figure 4:**
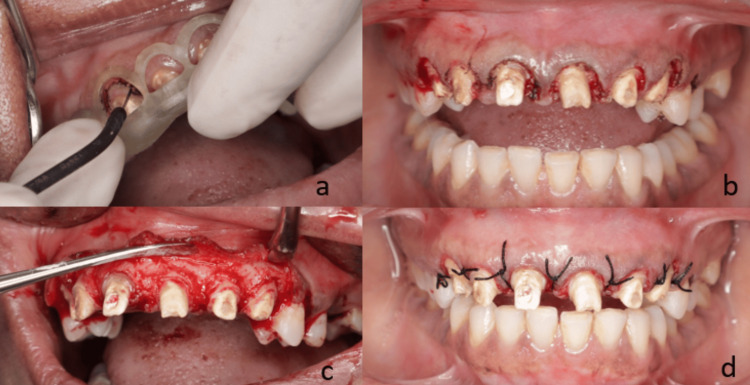
(a) Initial incision was made using electrocautery to ensure precise margins, with the surgical stent kept in place. (b) Soft tissues were debrided using Gracey curettes, and a crevicular incision was made with a scalpel. (c) A full-thickness flap was raised, and osteoplasty was performed using a round bur under copious saline irrigation. (d) Interrupted sutures with 4-0 Mersilk were placed to secure the flap

Under 2% local anesthesia with 1:200000 adrenaline, the surgical stent was placed, and electrosurgery was used to perform the precise initial external gingival incision according to the digital design (Figure 3). This was followed by a crevicular incision using a scalpel to raise a full-thickness flap. The gingival collar was excised using Gracey curettes, and an osteotomy was performed after measuring approximately 1.5 mm from the surgical guide margin to the alveolar crest. A distance of approximately 3 mm between the osseous crest and the gingival margin was maintained, measured using a UNC probe, to preserve the biologic width and ensure optimal conditions for prosthetic restoration. Osseous recontouring was performed using rotary instruments (carbide and diamond burs) with copious irrigation to prevent thermal injury and bone necrosis. The flaps were repositioned and secured using 4-0 Mersilk interrupted sutures (Figure 4). A provisional prosthesis was delivered on the same day to maintain aesthetics and function. Postoperatively, the patient was prescribed anti-inflammatory medication twice daily for three days and antibiotics (amoxicillin 500 mg) three times daily for seven days. Sutures were removed one week after the surgical procedure. The patient did not report any discomfort or postoperative complications.

**Figure 5:**
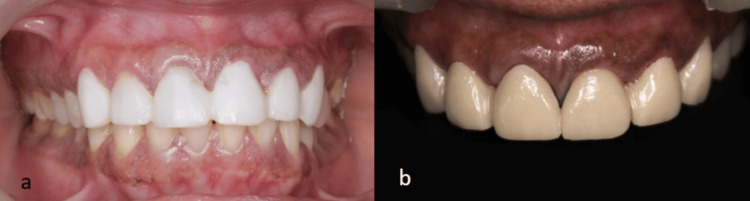
Postoperative images: (a) temporary prosthesis from tooth 13 to 23 following suture removal, and (b) definitive prosthesis from tooth 13 to 23 made of lithium disilicate, cemented after the healing period

**Figure 6:**
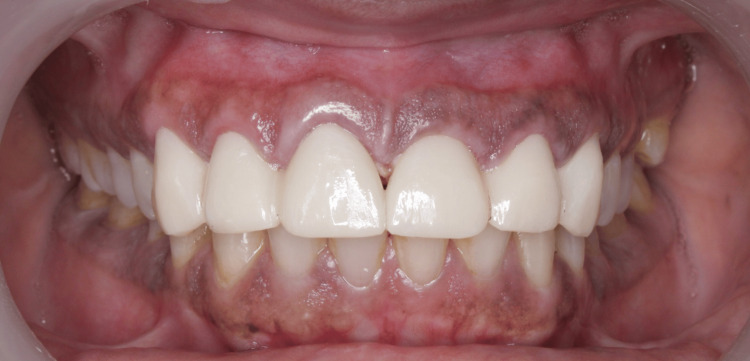
Six-month postoperative follow-up showing 0.5 mm gingival display, intact prosthesis, and healthy periodontium

Following an eight-week healing period, definitive full-coverage prostheses fabricated from lithium disilicate were cemented (Figure 5). Upon cementation, a comprehensive evaluation was performed to assess occlusal harmony and aesthetic outcomes, including tooth morphology, shade, and the establishment of appropriate proximal contact relationships with adjacent dentition. The patient was subsequently followed for six months to evaluate functional integration, periodontal response, and overall clinical performance of the restorations. Stable and healthy gingival margins with an intact prosthesis were observed clinically and radiographically (Figure 6).

## Discussion

Modern dentistry addresses the patient’s aesthetic needs as well as the functional and overall health of the oral cavity. In the present case, the patient was dissatisfied with her smile and therefore had both aesthetic and functional concerns. For her altered passive eruption, aesthetic crown lengthening was advised to enhance smile aesthetics and provide adequate restorative margins for anterior prostheses. Biologic width is essential for maintaining overall periodontal health, which directly influences tooth health. Violation of this dimension, often due to improperly placed restorative margins, can result in various complications. To prevent such issues, procedures such as surgical crown lengthening or orthodontic approaches may be utilized to maintain the integrity of the biologic width. In the present case, surgical crown lengthening with osseous reduction was performed to preserve the biologic width. This resulted in a significant increase in the visible crown length of the maxillary anterior teeth, accompanied by an adequate reduction in gingival display during smiling. On average, gingival exposure decreased by approximately 2 mm, which substantially reduced the patient’s excessive gingival display to 0.5 mm during her maximum smile. This was measured clinically by marking the distance between the lip margin and the gingival margin using digital software and confirmed by the mock-up.

For the success of aesthetic cases, precise evaluation and treatment planning are required. With the advent of newer digital technologies, it has become easier to plan mock-ups and anticipate future gingival margins; a digital surgical stent can be prepared to facilitate smooth surgical procedures. Studies have reported improved accuracy in achieving planned gingival margins and bone reduction, especially for inexperienced surgeons [9,10]. From a restorative and periodontal perspective, aesthetic crown lengthening plays a critical role in establishing a stable, healthy, and functional dentogingival complex. Facilitating appropriate margin placement and improving access for restorative procedures enhances marginal adaptation and restorative success. Aesthetic crown lengthening is an important procedure because improper margin placement, particularly in subgingival or intrasulcular locations with limited attached gingiva, has been associated with adverse periodontal outcomes. These include poor plaque control, inflammation, increased probing depth, bleeding on probing, gingival margin recession, and attachment loss. These considerations highlight the necessity of integrating the periodontium and biologic width into restorative planning. When indicated, osseous surgery and soft tissue augmentation should be performed to maintain both health and aesthetics.

Recent developments have greatly improved the accuracy of aesthetic outcomes and the convenience of performing the procedure in digital dentistry. Digital smile design, diagnostic mock-ups, and enhanced communication among the clinician, patient, and laboratory enable more precise treatment planning and outcome management. Additionally, these advancements facilitate the fabrication of restorative materials with superior mechanical and optical properties, enabling optimal aesthetic outcomes. Meanwhile, digital workflows may increase efficiency and reduce the uncertainty associated with manual procedures.

Liu et al. conducted a study comparing conventional and digitally driven aesthetic crown lengthening procedures. They concluded that the test group using a digital stent showed significantly better results, including less alteration in gingival margins, improved marginal fit, and higher red and white aesthetic index scores at six months compared to the conventional group [11]. Similar findings were reported in another study, in which patients treated with a digital stent demonstrated better gingival contours and more time-efficient treatment than conventional methods [12].

Certain limitations should be acknowledged. This is a case report of a single patient with no comparator. It is challenging to evaluate the stability of clinical and patient-reported outcomes over time in the absence of long-term follow-up data. Larger, controlled trials are required to confirm these findings and to compare conventional crown lengthening with modified surgical techniques, such as flapless or minimally invasive approaches, across diverse patient populations [13-17].

Overall, the findings of this study suggest that, in cases of altered passive eruption, aesthetic crown lengthening is an effective treatment for improving clinical parameters and patient satisfaction. Additionally, it emphasizes the importance of a patient-centered approach in aesthetic dentistry, recognizing that treatment success is ultimately determined not only by objective measurements but also by the patient’s perception of their smile.

## Conclusions

Digital technologies have facilitated shared decision-making, the use of visual aids, and realistic discussions of outcomes by aligning clinical goals with patient priorities. They may reduce operative time and the possibility of measurement errors, thereby helping practitioners achieve improved outcomes. In addition to satisfactory aesthetic results as reported by the patient, she did not experience any postoperative complications such as swelling, bleeding, or pain. During regular follow-ups over six months, satisfactory gingival healing was observed, as evidenced by gingival margin stability, absence of bleeding on probing, and stable probing pocket depths.

In the present case, a digitally guided crown lengthening procedure facilitated restorative therapy and improved aesthetic outcomes. Therefore, an accurate, interdisciplinary diagnostic approach, along with patient-centered care, is essential, as treatment should be guided by the patient’s concerns, expectations, and perception of their smile, as well as morphological criteria.
